# Integrated Prosthodontic Approach for Ectodermal Dysplasia: Tooth-Supported Overdenture and Implant-Supported Hybrid Prosthesis

**DOI:** 10.7759/cureus.111071

**Published:** 2026-06-18

**Authors:** Aishwarya Saini, Rekha Gupta, Shubhra Gill, Swarnim Singh, Jeldi Kusuma

**Affiliations:** 1 Prosthodontics, Maulana Azad Institute of Dental Sciences, New Delhi, IND

**Keywords:** digital work flow, ectodermal dysplasia, full mouth rehabilitation, implant-supported prosthesis, telescopic overdenture

## Abstract

Ectodermal dysplasia (ED) is a hereditary disorder characterized by defects in ectodermally derived structures such as teeth, hair, nails, and sweat glands. Oral manifestations, such as hypodontia, microdontia, and underdeveloped ridges, pose significant prosthodontic challenges. This case report describes the comprehensive rehabilitation of a patient diagnosed with ED. Clinical and radiographic evaluation revealed multiple missing teeth and reduced vertical dimension. A multidisciplinary plan was executed involving a tooth-supported telescopic overdenture in the maxilla and an implant-supported overdenture in the mandible, using digital planning and a 3D-printed surgical guide for implant placement. Occlusal rehabilitation was based on the shortened dental arch concept, ensuring functional efficiency and occlusal stability. The final prostheses restored esthetics, function, and comfort, with improved masticatory performance and patient satisfaction. This case highlights the effectiveness of a prosthodontically driven, digitally guided approach in managing ED-associated full mouth rehabilitation.

## Introduction

Full mouth rehabilitation is a comprehensive dental treatment approach aimed at restoring function, esthetics, and comfort in patients with multiple missing, malformed, or nonfunctional teeth. It often requires a multidisciplinary approach involving prosthodontics, implantology, and restorative dentistry to address both structural and functional deficits. The goal is not only to reestablish occlusion and mastication but also to improve phonetics, facial harmony, and overall quality of life. In complex cases, especially those involving congenital anomalies, the treatment must be individualized and staged to accommodate anatomical limitations and patient-specific needs [1].

Ectodermal dysplasia (ED) refers to a group of inherited disorders affecting ectodermally derived structures, including skin, hair, nails, sweat glands, and teeth. Over 180 subtypes exist, with varied inheritance patterns including X-linked, autosomal dominant, and autosomal recessive. The most common form, hypohidrotic ectodermal dysplasia (HED), often affects males more severely due to its X-linked recessive nature [2].

Dental anomalies are hallmark features of ED and include hypodontia, anodontia, microdontia, and peg-shaped teeth. These often lead to underdeveloped alveolar ridges, reduced vertical dimension of occlusion (VDO), and compromised facial esthetics. Such defects impair mastication, speech, esthetics, and psychosocial development [3].

Early diagnosis and intervention are critical in managing ED. Pediatric patients often require interim prosthetic solutions to support normal facial growth and improve function and appearance. Prosthodontic rehabilitation in ED patients demands a multidisciplinary approach. Treatment planning must be individualized, taking into account the patient’s age, growth potential, bone availability, and psychological readiness. Removable partial dentures or complete dentures may be used initially, with implant-supported prostheses considered later when skeletal growth is complete. Cone-beam computed tomography (CBCT) is essential for evaluating bone volume and planning implant placement, particularly in the mandibular interforaminal region, where bone tends to be more favorable. Advances in digital dentistry, such as computer-aided design (CAD) and computer-aided manufacturing (CAM) technology, 3D-printed surgical guides, and precision attachment systems, have significantly improved the predictability and success of prosthetic outcomes. In cases with partial dentition, telescopic overdentures offer a viable solution by utilizing remaining teeth for support and retention. These systems enable better access to hygiene and long-term maintenance. For edentulous arches, implant-supported hybrid prostheses provide stability and effectively restore function [4].

This case report describes the comprehensive prosthodontic management of a 21-year-old woman patient with features suggestive of ectodermal dysplasia, rehabilitated with a tooth-supported overdenture in the maxillary arch and an implant-supported hybrid prosthesis in the mandibular arch, at a restored vertical dimension of occlusion (VDO).

## Case presentation

**Figure 1:**
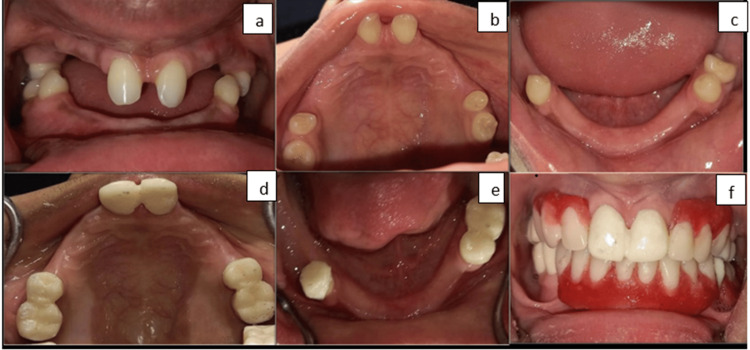
Diagnostic and transitional phase. (a) Frontal view showing peg-shaped incisors and missing upper and lower teeth; (b) and (c) occlusal views of partially edentulous in both arches; (d)-(f) provisionals are given at vertical dimension.

This case presents a 21-year-old woman patient who reported to the department with a chief complaint of multiple missing teeth in both her upper and lower jaws. The patient expressed concerns regarding impaired esthetics and compromised mastication. Her general medical history was non-contributory; however, the family history revealed similar dental anomalies among close relatives, suggesting a possible hereditary etiology. Based on clinical findings and the positive family history, a diagnosis of ectodermal dysplasia was made. This condition is a developmental disorder characterized by abnormal development of ectodermal structures, commonly resulting in oligodontia or anodontia, and often requiring early prosthetic rehabilitation. On intraoral examination, multiple teeth were found to be congenitally missing, and peg-shaped maxillary central incisors (11 and 21) were observed (Figure 1a). The missing teeth in the maxillary arch included 12, 13, 14, 22, 23, and 24, while in the mandibular arch, teeth 31, 32, 33, 34, 36, 41, 42, 43, and 44 were absent (Figures 1b, 1c). The patient also demonstrated a reduced vertical dimension of occlusion (VDO) and underdeveloped alveolar ridges. It was decided to increase the vertical dimension by 4 mm. A diagnostic removable partial denture and provisional crowns were fabricated and placed on the remaining permanent teeth to assess function and esthetics at the proposed VDO (Figures 1d-1f).

**Figure 2:**
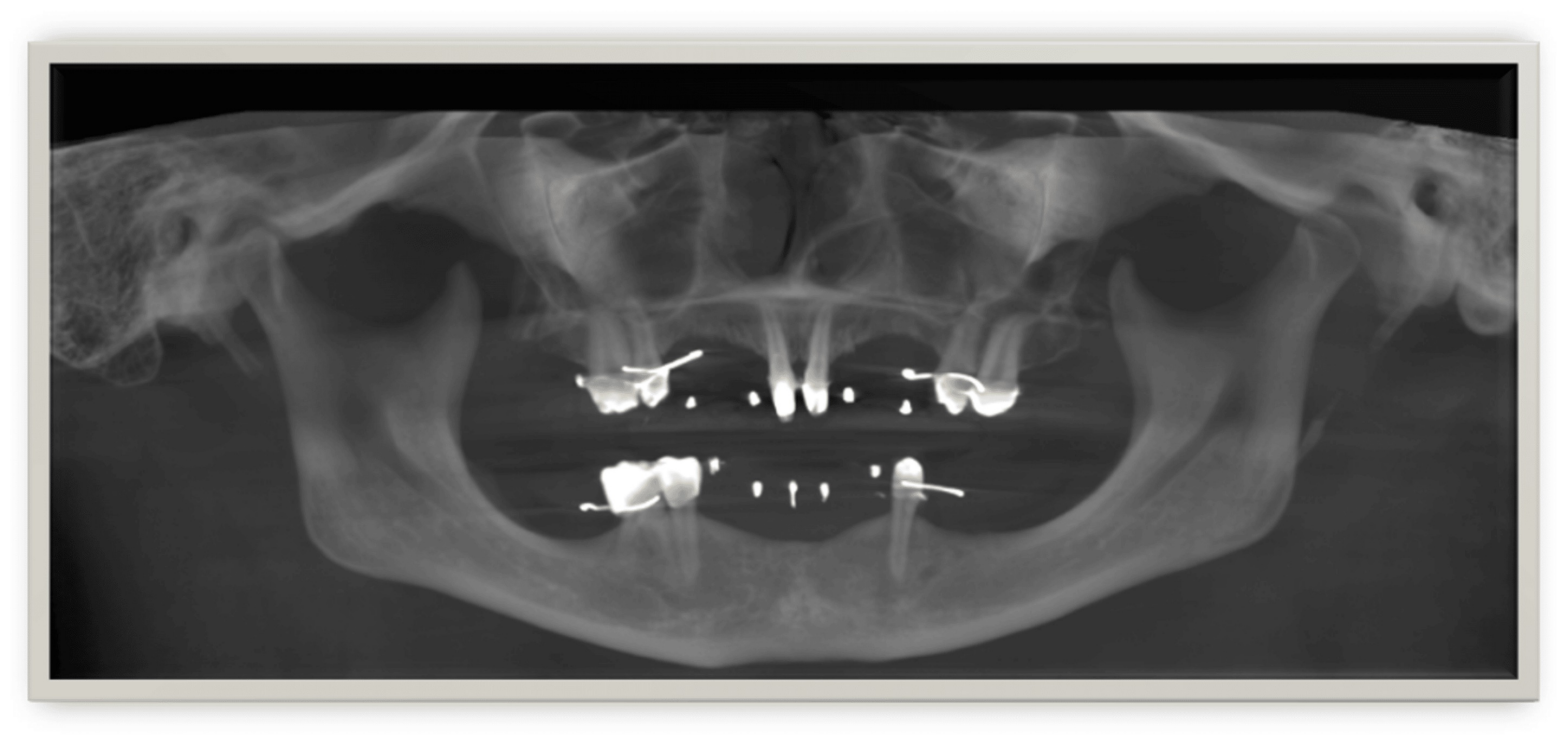
Panoramic view of the patient.

A thorough radiographic examination was conducted using panoramic radiography and cone-beam computed tomography (CBCT) to evaluate the existing bone morphology (Figure 2). The CBCT analysis confirmed that the anterior region of the mandibular arch exhibited sufficient bone width and height to support endosseous implants. Consequently, the treatment plan involved fabricating an implant-supported hybrid prosthesis in the mandible. To ensure precise implant placement, a 3D-printed surgical guide was designed based on a provisional prosthesis containing radiopaque markers placed at strategic positions. This radiographic stent was used to transfer the planned implant positions from the radiograph to the surgical field.

**Figure 3:**
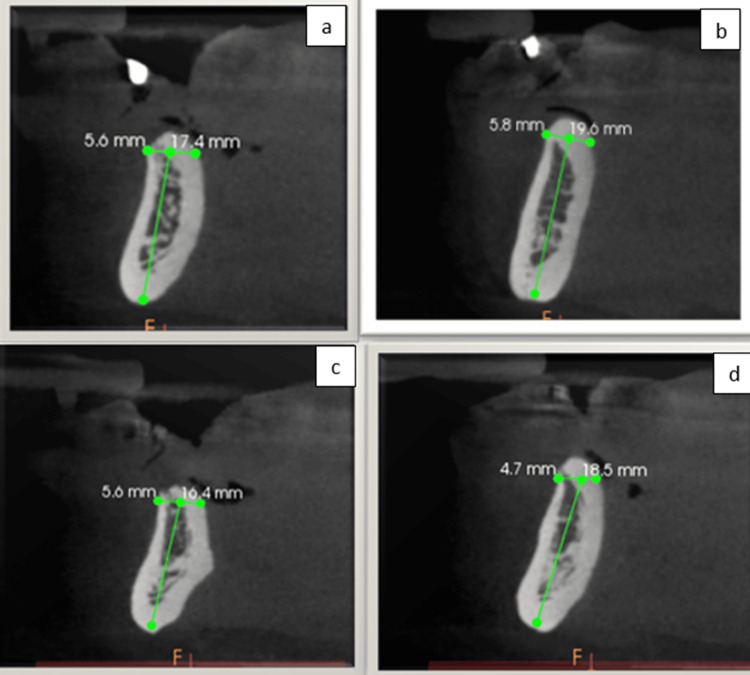
Sagittal sections of potential implant sites of mandibular bone in the interforamin region. (a) Site 44, (b) site 42, (c) site 34, and (d) site 32.

The mandibular arch was evaluated for four implant positions, designated as sites 44, 42, 32, and 34. The bone dimensions at these sites were as follows: site 44-17.4 mm height, 5.6mm width; site 42-19.6 mm height, 5.8 mm width; site 34-16.4 mm height, 5.6 mm width; and site 32-18.5 mm height, 4.7 mm width (Figure 3). The implant system selected for this procedure was the Osstem TSIII (Osstem Implant Co., Ltd., Seoul, South Korea), with 3.5-mm-diameter, 10-mm-long implants for all five sites. Surgical planning was meticulous and aimed to avoid vital anatomical structures such as the mental foramen and inferior alveolar nerve. A digitally designed, 3D-printed surgical guide was fabricated using CBCT analysis and prosthetic planning.

**Figure 4:**
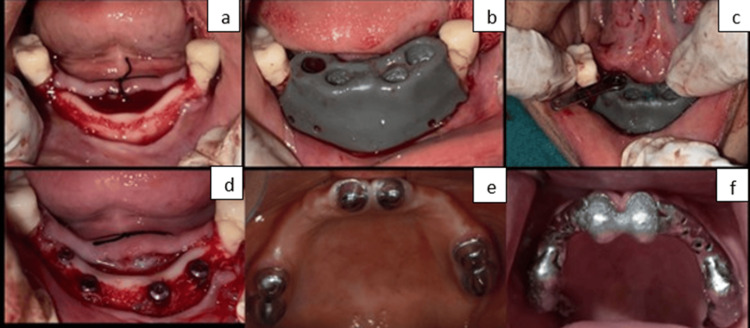
Intra-operative phase. (a) Flap incision was given, reflected from the 34 to 44 region; (b) and (c) printed surgical guide was placed and fixed; (d) cover screws were placed after implant placement; (e) primary copings are given for maxillary over denture; (f) metal framework for telescopic overdenture checked.

On the day of surgery, the patient was anesthetized using bilateral inferior alveolar nerve blocks, lingual nerve blocks, and mental nerve blocks to ensure adequate analgesia. A crestal incision was made in the mandibular anterior region, supplemented by vertical releasing incisions in relation to teeth 35 and 45. A trapezoidal full-thickness mucoperiosteal flap was carefully reflected to expose the alveolar ridge (Figure 4a). A 1.5 mm osteoplasty was performed using a round bur under copious saline irrigation to flatten the ridge and enhance the fit of the surgical guide. The surgical guide was then secured in place with retentive screws, ensuring stable and accurate positioning during osteotomy preparation (Figures 4b, 4c).

The osteotomy sequence began with a pilot drill, followed by a Lindemann drill, with direction and angulation verified using paralleling pins and intraoperative radiographs. Subsequent enlargement of the osteotomy was done using twist drills of 2.2 mm and 3.0 mm diameter to the planned depth of 10 mm. Final positioning was verified radiographically before implant placement. The implants were placed using a no-mount fixture driver, and torque was manually applied with a torque wrench to ensure primary stability. Cover screws were placed over each fixture (Figure 4d). The surgical site was irrigated thoroughly with normal saline and betadine, and the flap was approximated and sutured using simple interrupted sutures.

**Figure 5:**
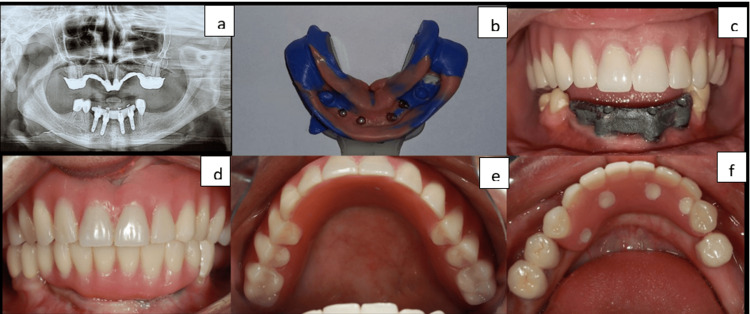
Prosthetic phase. (a) Orthopantograph of osseointegrated implants; (b) open tray impression; (c) metal framework checked; (d) final prosthesis in occlusion; (e) and (f) maxillary telescopic overdenture and mandibular hybrid prosthesis.

Post-operatively, the patient was provided with a provisional prosthesis (relieved partial denture) fabricated with a raised vertical dimension to facilitate adaptation and assess functional outcomes before proceeding with the final prosthesis. This prosthesis also served as a reference for esthetics, phonetics, and occlusion. The provisional phase was essential in reestablishing facial proportions, lip support, and occlusal balance, which are often altered in patients with ectodermal dysplasia. After a healing period of four months and confirmation of successful osseointegration (Figure 5a), a hybrid implant-supported prosthesis was fabricated to span the 44-34 region (Figures 5d, 5f). Porcelain-fused-to-metal (PFM) crowns were placed on teeth 45, 46, and 35. In the maxillary arch, the remaining teeth (11, 21, 15, 16, 25, and 26) were prepared to receive primary and secondary copings (Figure 5e), over which a tooth-supported overdenture was fabricated. A direct metal laser sintered (DMLS) cobalt-chromium framework was fabricated (Figure 5f), on which a telescopic denture was constructed and delivered (Figure 5e).

Occlusal contacts were verified and adjusted intraorally to ensure balanced occlusal contacts. The patient was instructed on hygiene maintenance using interdental brushes and periodic disinfection of the prosthesis. Follow-ups at regular intervals assessed tissue response, prosthesis fit, and implant health. The patient showed good adaptation, improved esthetics, and enhanced masticatory efficiency, indicating successful rehabilitation.

## Discussion

In this case, the diagnosis of ED was based on clinical and radiographic findings, including congenitally missing teeth, peg-shaped incisors, and reduced alveolar bone volume. Although genetic testing was not performed, the familial pattern and phenotypic presentation strongly supported the diagnosis.

Restoration of VDO was a critical first step. It was achieved incrementally, using a diagnostic removable partial denture and provisional crowns to assess the patient's adaptation. This strategy aligns with established protocols that emphasize gradual reestablishment of occlusal parameters to avoid temporomandibular discomfort and ensure neuromuscular adaptation [5].

In the maxillary arch, limited bone and strategic positioning of remaining teeth made the patient a suitable candidate for a telescopic overdenture. The use of direct metal laser sintered (DMLS) cobalt-chromium frameworks and primary copings ensured a precise fit and facilitated hygiene maintenance. Telescopic systems offer superior retention and long-term prognosis in partially edentulous ED patients, especially when conventional dentures are insufficient. Overdentures provide a more retentive option and are used when teeth are present for support. They help preserve the alveolar bone compared with complete dentures, as shown by Gupta et al., who found a significant reduction in alveolar bone loss in the overdenture group after two years. The only downside is that overdentures require aggressive tooth preparation and elective endodontic treatment of otherwise healthy teeth [6].

Although some clinicians express concern about implant placement in ED patients under 18 years, evidence supporting a high risk of failure is limited. The anterior region of the mandible is considered transversely stable by the age of six years, and careful site selection in the posterior mandible can mitigate complications related to lingual plate remodeling. In contrast, transverse growth of the maxilla continues into the second decade, making early implant placement in that region less advisable [4]. Therefore, when planning osseointegrated implants in ED patients, dental and skeletal maturity, not chronological age, must guide timing and site selection to avoid infraocclusion of the prosthesis. In this case, implant placement in the mandibular interforaminal region was facilitated by adequate bone volume. The resulting hybrid prosthesis, supported by four implants, provided excellent stability and function. Hybrid prostheses are particularly beneficial in ED cases due to their rigid support and ability to compensate for soft tissue deficiencies [7,8].

To optimize prosthetic design and accommodate anatomical limitations, the shortened dental arch (SDA) concept was incorporated. Originally proposed by Kayser, SDA advocates for functional restoration using a reduced number of occluding pairs, typically extending to the second premolars. In this case, the hybrid prosthesis restored function up to the premolar zone, avoiding posterior implant placement in areas with limited bone volume. SDA has been shown to maintain satisfactory oral health-related quality of life and masticatory efficiency, especially when anterior guidance and occlusal stability are preserved [9].

This case underscores the importance of a multidisciplinary approach; the combination of implant-supported and telescopic prostheses allowed restoration of esthetics, speech, and masticatory efficiency while preserving the remaining dentition and optimizing hygiene.

## Conclusions

This case demonstrates successful rehabilitation of ectodermal dysplasia through a multidisciplinary approach, including incremental VDO restoration, a maxillary telescopic overdenture, and a mandibular implant-supported hybrid prosthesis. At the three-year follow-up, the implants exhibited satisfactory stability, with acceptable probing depths and no signs of peri-implant pathology, supporting the favorable clinical outcome of the treatment. The shortened dental arch concept enabled functional outcomes despite anatomical limitations, highlighting the importance of individualized, growth-based treatment planning for predictable results. However, as this is a single case with a limited follow-up period, longer-term evaluation is necessary before establishing the long-term predictability of this treatment approach.
